# Material Modeling of PMMA Film for Hot Embossing Process

**DOI:** 10.3390/polym13193398

**Published:** 2021-10-02

**Authors:** Dongwon Yun, Jong-Bong Kim

**Affiliations:** 1 Department of Robotics Engineering, Daegu Gyeongbuk Institute of Science and Technology, 333 Techno jungang-daero Hyeonpung, Dalseong-gun, Daegu 42988, Korea; mech@dgist.ac.kr; 2 Department of Mechanical and Automotive Engineering, Seoul National University of Science and Technology, 232 Gongneung-ro, Nowon-Gu, Seoul 01811, Korea

**Keywords:** constitutive model, embossing, finite element method, Poly methyl methacrylate, polymer

## Abstract

This study provides an analysis of the hot embossing process with poly methyl methacrylate (PMMA) film. The hot embossing process engraves a fine pattern on a flexible film using a stamp, applied heat and pressure. As the quality of the embossing pattern varies according to various process variables, the mechanism of making the embossed shape is complicated and difficult to analyze. Therefore, analysis takes much time and cost because it usually has to perform a lot of experiments to find an appropriate process condition. In this paper, the hot embossing process was analyzed using a computational analysis method to quickly find the optimal process. To do this, we analyzed the embossing phenomenon using the finite element method (FEM) and arbitrary Lagrangian–Eulerian (ALE) re-mesh technique. For this purpose, we developed a constitutive model considering the strain, strain rate, temperature-dependent stress and softening of the flexible film. Work hardening, strain softening, and temperature-softening behavior of PMMA materials were well described by the proposed method. The developed constitutive model were applied in the embossing analysis via user-subroutine. This proposed method allowed a precise analysis of the phenomenon of film change during the hot embossing process.

## 1. Introduction

The hot embossing process is a technique used to engrave micro or nano-sized patterns onto a flexible substrate. Recent studies have demonstrated that this process is much simpler and generates less waste than silicon-based conventional semiconductor manufacturing methods. Hot embossing typically involves transferring a pattern by contacting a finely patterned stamp with a flexible substrate such as a polymer film while applying heat and pressure to the flexible substrate. Among various applications, the hot embossing process allows low cost mass production of single-use plastic microfluidic devices [1]. Roll-to-roll hot embossing has also been studied to increase the speed of embossing production using micro or nano patterning [2]. Nanoimprinting by melt processing (NIMP) was developed to overcome the limitations of the nano imprint lithography (NIL) process, and to achieve high-quality imprints at low temperature and pressure [3]. So far, most applications have been in the biomedical field, with an emphasis on chip-type micro devices [4]. However, recently, the range of hot embossing applications has diversified. For example, arrays of nanofibrils have been obtained using a hot-embossing thermo-plastic material as an alternative to typical methods like silicon or silicon dioxide dry-etching technologies, or the deposition of nickel or other metals on patterned resist substrates (the lithographie, galvanoformung, abformung (LIGA) process) [5]. A transparent and flexible nano-pattern was fabricated using the thermoplastic properties of fluorinated ethylene propylene (FEP) to build an energy-harvesting device.

In the hot embossing process, the major factors are pressure, temperature and time [6]. The quality of the patterns generated by this process are greatly affected by various conditions such as the film speed, temperature and pressure. In addition, since the behavior of the film also changes from an elastic region to a plastic region, it is difficult to theoretically predict or calculate in advance the amount of deformation produced by the process. For this reason, many studies have had to focus on finding process conditions suitable for each film and pattern, using multiple experiments. For example, Lan et al. [6] conducted hot embossing experiments and found which parameter played the most prominent part. Experiments were planned and conducted based on Taguchi’s numerical methods to find optimized control process variables for the roll-to-roll hot embossing process [7].

Numerical analyses of the hot embossing process have also been performed to develop a computational method which can reduce the process time and effort, by determining the influence of each parameter on the embossing process. Numerical simulations of the embossing process were also carried out to compare with observed polymer flow patterns, and to investigate the effect of boundary conditions and air entrapment on imprinting quality [8].

However, the real embossing process is more complicated, since it involves transitions between a solid like state or fluid like state depending on the temperature. For this reason, many research groups have used commercial software for more precise results. For example, the filling process of a micro prism array by isothermal hot embossing in solid-like state (IHESS) with different mold temperature, pressure, and holding time were simulated by DEFORM to cut the cycle time [9]. DEFORM was also used for numerical models for hot embossing to analyze the advance of the flow front of the molten polymer [10]. To consider the different phenomena between molding and demolding, two kinds of software have been used simultaneously. For mold filling, Moldflow was applied and for demolding, ANSYS was used to describe the typical damage of the replicated microstructures [11]. A mathematical representation in MARC code was used to simulate the hot embossing of a polymer material [12]. Another paper described the application of contact-stress analysis to understand the mechanism when using molds with micro-features to hot-emboss PMMA substrates [13]. A finite element analysis was conducted to simulate the deformation behavior of polymer during the hot embossing process, and it was revealed by analysis and experiment that swallowtails were the result of stress concentration and plastic deformation [14]. A study on the hot embossing process for forming a microstructure for a drug delivery system was conducted. Modeling of the time-dependent relaxation modulus of the material was built for the analysis, and using the commercial software ABAQUS, FE analysis on deformation during the hot embossing processing was also performed [15].

In this study, a study on the hot embossing process for PMMA film was conducted. First, in order to model material properties which depend on various conditions such as strain, strain rate, and temperature, material modeling was performed in more detail than in previous studies. In order to obtain an accurate deformed shape, an accurate material model should be used. To this end, it was assumed that the flow stress of a material depends on stress, temperature, strain, and strain rate. A new constitutive model for PMMA were developed considering work hardening, strain rate hardening, and strain and temperature softening. Next, using this modeling, finite element analysis was performed by applying it to ABAQUS via user-subroutine, a commercial finite element analysis software, and the developed modeling and analysis results were verified by comparison with the experimental results. Finally, based on the developed model and finite element analysis method, a simulation of the hot embossing process for the PMMA film, which is the purpose of this study, was performed, and the deformation shape, stress, and temperature change of the material according to the amount of pressing of the stamp could be calculated.

## 2. Materials and Methods

### 2.1. Proposed Modeling Method

**Figure 1:**
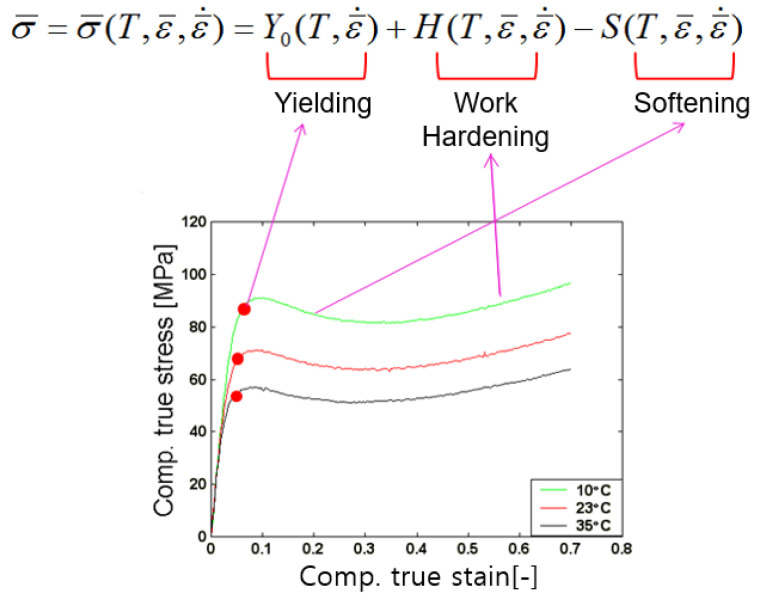
Stress-strain relation of poly methyl methacrylate (PMMA) in the low temperature region.

In this study, we analyzed the hot embossing process for PMMA film. In order to perform the analysis, first, it was necessary to accurately describe the flow stress for wide ranges of temperature, strain, and strain rate. Under the assumption that a plastic deformation analysis is more accurate than a fluid flow analysis at the forming temperature of 120 °C or less, the flow stress was described. Experimental data about the stress-strain relation for various temperatures and strain rates were obtained from the work of Baselmans et al. [16]. From this data, it could be seen that as the strain rate increases, the flow stress increases, and as the temperature increased, the flow stress decreased. In addition, the relationship between strain and flow stresses was very nonlinear, and it was clear that a prediction of the embossing load may have included a large error if such characteristics are not considered in the finite element analysis of the hot embossing process. However, the references providing this data did not provide data on flow stresses at temperatures above 100 °C. As in the hot embossing process for PMMA films, temperatures between about 100 °C and 120 °C are important, flow stress data above 100 °C was subsequently acquired from the work of Ghatak and Dupaix [17]. To use the data given in the above references for analysis, it was necessary to formulate the data to represent the relationship between flow stress, temperature, strain, and strain rate. For this purpose, we assumed the following flow stress,$\;\bar{\sigma };$

(1) $$\bar{\sigma }=\bar{\sigma }\left(T,\;\bar{\varepsilon },\;\dot{\bar{\varepsilon }}\right)$$

where T, $\bar{\varepsilon },\;$ $\dot{\bar{\varepsilon }}$ are temperature, strain and strain rate respectively. The flow stress curve of PMMA shows a very different tendency than metals such as steel or aluminum. For example, metal only shows work hardening with increasing strain rate, however, PMMA exhibits softening at a certain region with increasing strain, which is different from the softening property according to temperature. Therefore, the function is divided into three characteristic values as shown in Figure 1. Using the data in the reference [14], we have drawn this relationship. 

The yield point is assumed to be a function of temperature and strain rate, and work hardening and softening to be a function of temperature, strain and strain rate. As a result of comparing and analyzing the characteristics of various graphs, it was decided that the yield stress, work hardening, and softening should be composed of the sum or difference, rather than the product, as:

(2) $$\bar{\sigma }=\bar{\sigma }\left(T,\;\bar{\varepsilon },\;\dot{\bar{\varepsilon \;}}\right)=Y_{0}\left(T,\;\dot{\bar{\varepsilon \;}}\right)+H\left(T,\bar{\varepsilon },\;\dot{\bar{\varepsilon \;}}\right)-S\left(T,\bar{\varepsilon },\;\dot{\bar{\varepsilon \;}}\right)$$

The first term is related to yield, the second term is related to work hardening, and the last term is related to softening.

### 2.2. Modeling of Initial Yield Stress

**Figure 2:**
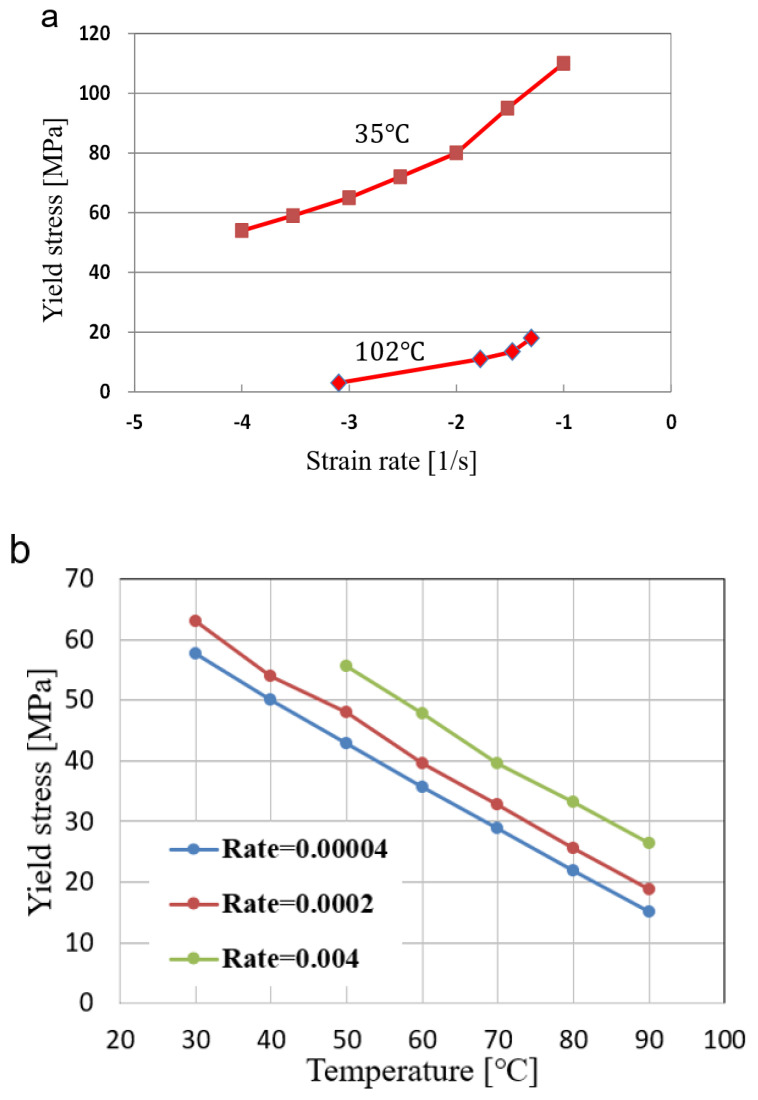
Yield stress variation with (**a**) temperature and (**b**) strain rate.

In this section, the change in the yield stress value is expressed as an equation. Figure 2 shows the yield stress according to temperature and strain rate. Yield stress was obtained from each of the references [16,17,18]. The yield stress for temperature were shown in Figure 2a. It was shown that the yield stress is almost linearly decrease as temperature in the range from 300 to 370 K. The graph at the specific temperature shows that it consists of two straight lines with different slope. This means that the strain rate-dependent behavior can be divided into two regions, i.e., a smaller and larger slope. It can be seen that there is a point where the two straight lines meet. The function for the yield stress is found by using the following procedure.

(a) determining the coefficients of two straight lines at a given temperature as a function of temperature;

(b) determining two straight lines at a given temperature using the coefficients of the line;

(c) determining the flow stress using the strain rate.

In this way, the function for yield stress can be expressed as the following equation. Considering Figure 2a, the linear dependency of yield stress for temperature is assumed.

(3) $$Y_{0}\left(T,\;\dot{\bar{\varepsilon \;}}\right)=\max\left[\left\{A_{11}+A_{12}\log_{10}\left(\dot{\bar{\varepsilon \;}}\right)\right\},\;\left\{A_{21}+A_{22}\log_{10}\left(\dot{\bar{\varepsilon \;}}\right)\right\}\right]$$

$$A_{11}\left(T\right)=-1.23457T+484.4627$$

$$A_{12}\left(T\right)=-0.09915T+43.53797$$

$$A_{21}\left(T\right)=-1.32881T+549.4722$$

$$A_{22}\left(T\right)=-0.06701T+50.62060$$

### 2.3. Modeling of Work Hardening

Next, we want to find an expression for terms related to work hardening. We tried to formulate the flow stress data related to the work hardening and the following relation was found.

(4) $$H\left(T,\bar{\varepsilon },\;\dot{\bar{\varepsilon \;}}\right)=H_{max}\left(T,\dot{\bar{\varepsilon \;}}\right)\frac{\exp\left(0.7\bar{\varepsilon }\right)-1}{\exp\left(0.49\right)-1}$$

(5) $$H_{max}\left(T,\dot{\bar{\varepsilon \;}}\right)=0.2\left(10+Y_{0}\left(T,\dot{\bar{\varepsilon \;}}\right)\right)$$

**Figure 3:**
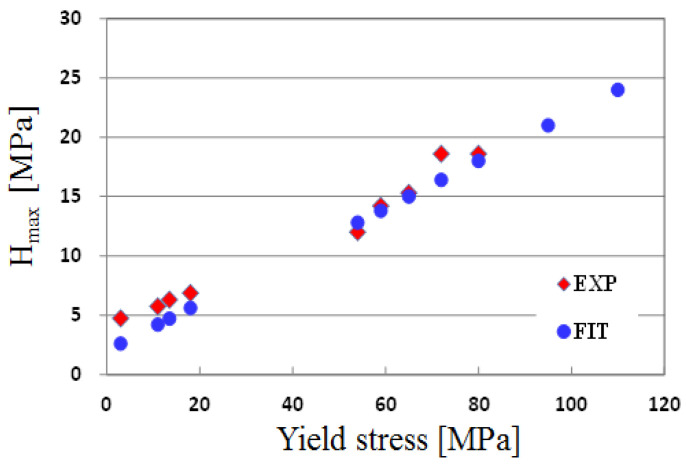
Relation between the maximum work-hardening and initial yield stress and fitted data using Equation (4).

The maximum hardening (H_max_) data were extracted from the data in the references [14,15] and plotted in Figure 3. After many trials to determine the relation between H_max_ with temperature and strain rate, we found that the H_max_ can be expressed as a function of initial yield stress, as in Equation (5). The predicted and experimental data are shown in Figure 3. From this figure, it was found that the work hardening value according to the change in the stress calculated by the obtained equation agreed well with the experimental results.

### 2.4. Modeling of Softening

In order to find the equation for the softening characteristics, fitting curves were also obtained for the data related to the softening provided in the references [16,17]. The softening is described from two viewpoints, temperature and strain softening, as follows:

(6) $$S\left(T,\bar{\varepsilon },\;\dot{\bar{\varepsilon \;}}\right)=S_{max}\left(T,\dot{\bar{\varepsilon \;}}\right)\left(1-\frac{1}{\exp\left(8\bar{\varepsilon \;}\right)}\right)$$

(7) $$S_{max}\left(T,\dot{\bar{\varepsilon \;}}\right)=Y_{0}\left(T,\dot{\bar{\varepsilon \;}}\right)\min\left[\left\{0.05+0.4\exp\left(0.5\log_{10}\left(\dot{\bar{\varepsilon \;}}\right)\right)\right\},0.7\right]$$

**Figure 4:**
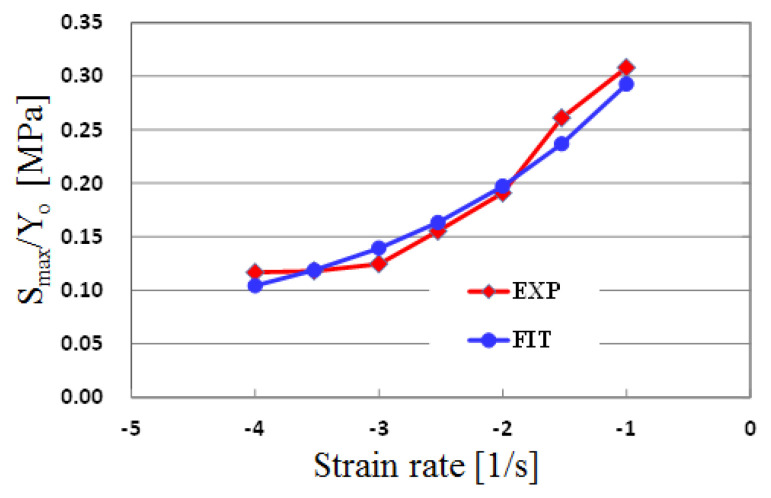
Maximum softening behavior for strain rate.

The maximum softening S*_max_* was extracted from the experimental data and plotted in Figure 4. The best fitting equation for the S_max_ in Figure 4 is proposed as Equation (7), and the fitted results are also shown in Figure 4. It was found that the obtained equation showed a similar tendency to the experimental results. The final number “0.7” in Equation (7) is an empirically used value to prevent stress from becoming negative.

## 3. Results and Discussion

### 3.1. Verification of the Proposed Constitutive Model

**Figure 5:**
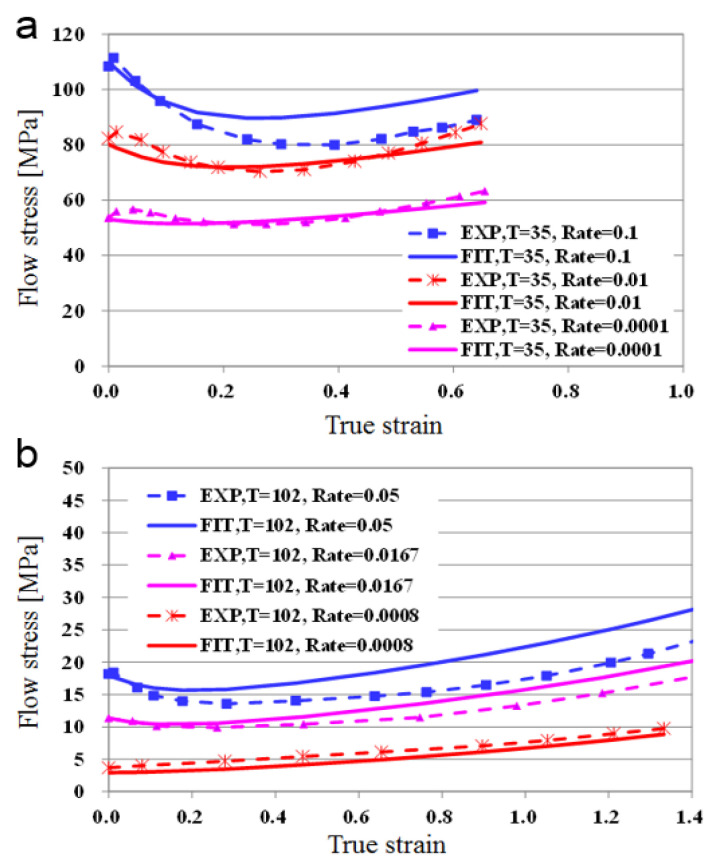
Fitting results at (**a**) low temperature (35 °C) and (**b**) high temperature (102 °C).

**Figure 6:**
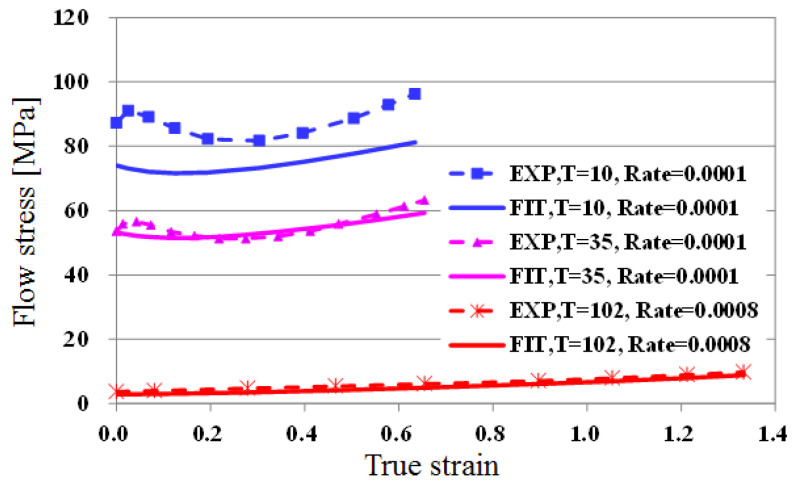
Fitting results for a wide range of temperatures at a low strain rate.

Figure 5a,b show the results of fitting at 35 °C in the low temperature region, and 102 °C in the high temperature region. For the cases of 0.1 and 0.05, where the strain rate is relatively large, there is some error in the fitted results, but they are in good agreement with experimental values in other areas. From this comparison, we conclude that the equation for the obtained flow stress can be used for finite element analysis. Figure 6 shows the results of fitting flow stress by temperature, when the strain rate is relatively small. There is a relatively larger difference at low temperatures. As can be seen in Figure 5 and Figure 6, the error is relatively large when temperature is low and strain rate is large. The interaction between temperature and strain rate may have an effect on flow stress, i.e., on the initial yield stress, hardening, and softening. In this study, however, the maximum hardening and softening amounts are described only as a function of the initial yield stress for simplicity of the proposed model. For a more accurate constitutive model, the interaction between temperature and strain rate should be considered. However, overall, the fitted value and the experimental value matched well. Therefore, the obtained formula can be utilized for the analysis of the embossing process according to temperature.

Based on the above results, the data fitting for flow stress was considered satisfactory, and the hot embossing analysis of the PMMA film was performed using the obtained flow stress equation.

### 3.2. Finite Element (FE) Modeling and Case Study

Next, a finite element analysis of the PMMA film was performed using the flow stress model for the material obtained from the previous section. ABAQUS was used for the analysis and a user subroutine was developed in order to use the obtained flow stress model.

In a large deformation analysis, “re-meshing” is required when elements are severely deformed. Abaqus provides an ‘ALE’ technique to overcome this severe deformation problem. In the hot embossing process, heat transfer takes place between the hot die and cool film during forming, and this heat transfer has a great effect on the forming quality. Accordingly, a transient heat transfer analysis needs to be performed together with the deformation analysis. Therefore, the ‘Explicit’ analysis was employed to predict the heat transfer and deformation together.

The overall equilibrium equation for explicit dynamic analysis is written as follows [19]:

(8) $$\frac{\partial }{\partial x}\cdot \sigma +\rho b=\rho \ddot{u},$$

where σ is the stress tensor, **b** is the body force vector, ρ is material density, and $\ddot{u}$ is the acceleration vector. Through the virtual work principle and finite element discretization, Equation (8) leads to:

(9) $$\ddot{u}=M^{-1}\cdot \left(F_{ext}-F_{int}\right)$$

In Equation (9), M is mass matrix, **F**_ext_ is external force vector, and **F**_int_ is internal force vector. For time integration, Equation (9) can be rewritten for current time step as:

(10) $${\ddot{u}}^{\left(i\right)}=M^{-1}\cdot \left(F_{ext}^{\left(i\right)}-F_{int}^{\left(i\right)}\right)$$

where the superscript (*i*) refers to time step increment number. The equations of motion for the body are integrated using the explicit central difference integration rule:

(11) $${\dot{u}}^{\left(i+\frac{1}{2}\right)}={\dot{u}}^{\left(i-\frac{1}{2}\right)}+0.5\left(∆t^{\left(i+1\right)}+∆t^{\left(i+1\right)}\right){\ddot{u}}^{\left(i\right)}$$

(12) $$u^{\left(i+1\right)}=u^{\left(i\right)}+∆t^{\left(i+1\right)}{\dot{u}}^{\left(i+\frac{1}{2}\right)}$$

where $\dot{u}$ and u are velocity and displacement vector, respectively. The superscript (*i+1/2*) and (*i − 1/2*) refer to mid-increment values. First, current step variables such as stress, strain, and external forces are calculated using displacements at current step. Using current step variables, acceleration is calculated by Equation (10). Then, velocity and displacement vectors at next step are calculated by Equations (11) and (12). These procedures are repeated until given time is reached.

**Figure 7:**
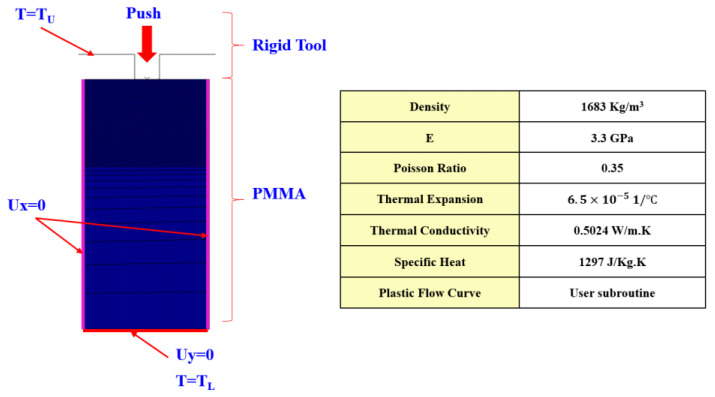
Analysis model and material properties.

The figure below shows a 2D model used for the analysis. Analysis was carried out for only one pattern by assuming symmetry. The upper stamp was modeled as a rigid body, and a constant temperature boundary condition was imposed for the heat transfer analysis. The lower plate was not modeled because the thickness of the film is sufficiently large compared to the depth of the patter, but a constant temperature condition was imposed for the lower edge of the PMMA film. The left and right sides of the film were assumed to be fixed in the direction of the *x* axis considering the model symmetry, and the bottom of the PMMA was fixed with zero displacement in the *y* direction considering the back plate. An initial temperature condition was applied for the PMMA film. The physical properties of the PMMA are shown in the table in Figure 7 [20,21]. The embossing time was 0.1 s.

In the hot embossing process, not just one pattern, but several patterns are formed at the same time. Also, the depth of the pattern to be formed tends to be different. It is possible to calculate the total load when several patterns are formed by accurately analyzing the depth-load curve for one pattern using the finite element method.

**Figure 8:**
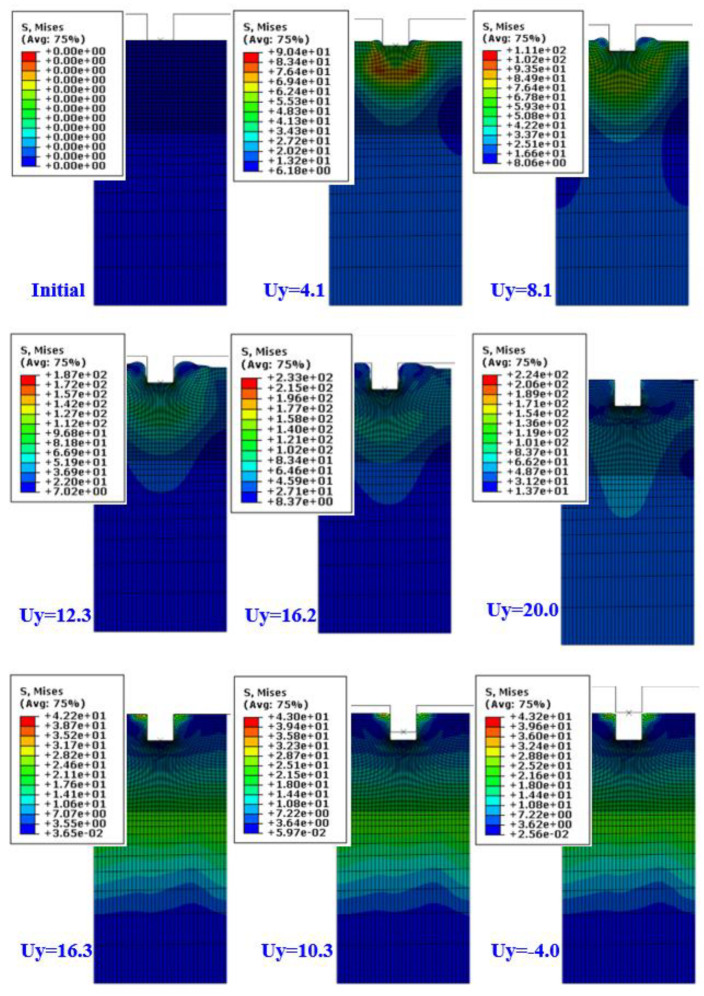
Deformed shape and von-Mises stress distribution (Tool: 100 °C, Sheet: 25 °C).

Using the constitutive model of the material obtained in the previous section, the analysis of the hot embossing process was carried out for a concave stamp at the stamp temperature (upper plate) of 100 °C and a lower plate temperature of 25 °C. The required load for molding and mold removal was calculated through the analysis, and the analysis results for stress inside the material according to the stamping depth are shown in Figure 8. The upper die moves up to 20 mm in 0.1 s. When the die displacement is 16.2 mm, most of the forming is completed. After that, volume contraction takes place due to the high die load. This is clearly shown in the deformed shape of unloading. When the die displacement is 16.3 mm (unloading), there is no clearance between the upper die and PMMA. This means that the volume has expanded during die displacement of 20 to 16.3 mm.

**Figure 9:**
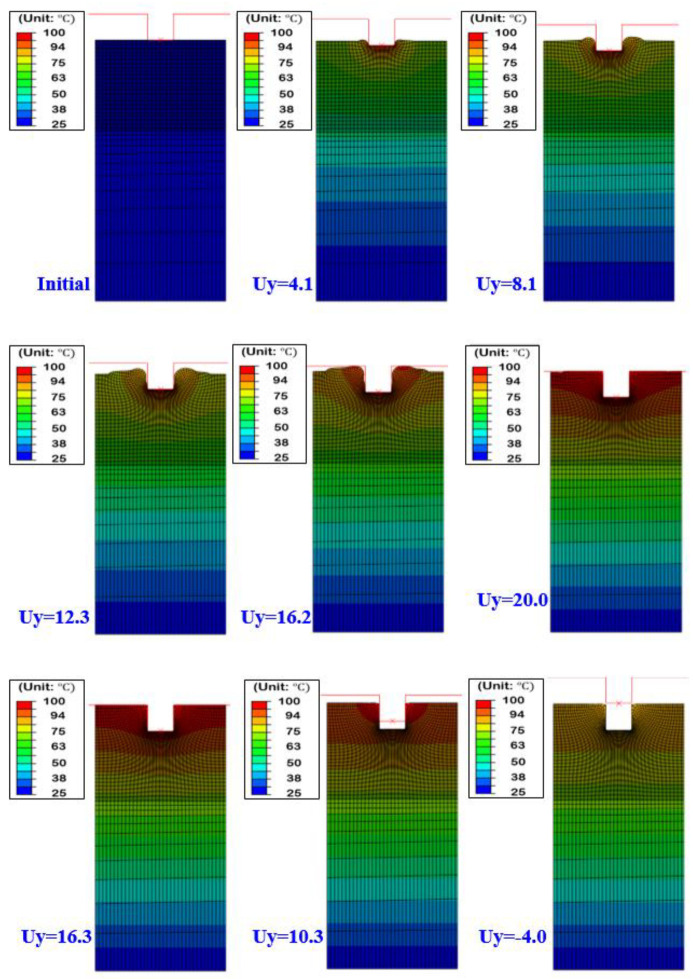
Deformed shapes and temperature distribution (Tool: 100 °C, Sheet: 25 °C).

Figure 9 shows the temperature distribution during forming and unloading. It can be seen that the temperature is transmitted from the upper die to the lower plate in the PMMA film. It was also found that the lower side of the film remained at almost the initial temperature even when the stamp completely penetrated the depth of the stamp. The temperature of the upper surface of the film was heated to the maximum stamp temperature of 100 °C and dropped to about 79 °C as soon as the stamp was removed from the film. As can be seen in the figure, the temperature of the PMMA at the contact region with the upper die is relatively higher than the middle region. Therefore, in Figure 8, the flow stress in the contact region is relatively lower than in the middle region due to the temperature softening. This shows that the constitutive model was successfully implemented in the analysis.

## 4. Conclusions

The hot embossing process was analyzed using the explicit finite element method. For the finite element analysis, first a constitutive model was developed using experimentally obtained stress–strain relationships for wide ranges of strain, strain rate, and temperature. The constitutive model was developed considering work hardening, strain rate effect, and strain and temperature softening. The constitutive model was verified using experimental data, and it was shown that the model can describe the experimental data for a wide range of strains, strain rates, and temperatures.

Finite element analysis of the hot embossing process was then carried out. The analysis was successfully carried out using the ALE remesh technique. The developed constitutive model was applied to the FE-analysis via user-subroutine. Analysis of the heat transfer from the die to the PMMA material and inside of the PMMA was also carried out together with the forming analysis. The deformed shape was successfully predicted. A constitutive model for PMMA and analysis method for hot embossing process using ALE technique and user-subroutine were established in the work. The finite element analysis method developed in this study can be used when designing the hot embossing process for any polymer film. By using the accurately developed constitutive model and analysis method, the temperature and forming load conditions for desired embossing pattern can be estimated before the actual equipment is manufactured, the manufacturing. As a result, the development cost of equipment can be lowered, and the development time also can be reduced. It is expected to be widely employed in the hot embossing process to be used in microfluidic devices, optical films, flexible devices, etc.

## Data Availability

The data presented in this study are available on request from the corresponding author.

## Abbreviations

ALE:	arbitrary Lagrangian–Eulerian
$\bar{\varepsilon }$:	Strain
$\dot{\bar{\varepsilon }}$:	Strain rate
FEM:	Finite element method
FEP:	Fluorinated ethylene propylene
H:	Hardness
LIGA:	Lithographie, Galvanoformung, Abformung
NIL:	Nano Imprint Lithography
NIMP:	Nanoimprinting by melt processing
PMMA:	Poly methyl methacrylate
S:	Softness
T:	Temperature
